# Developing a web‐based dashboard for adaptive radiotherapy workflows

**DOI:** 10.1002/acm2.70546

**Published:** 2026-04-06

**Authors:** Chloe DiTusa, Ara Alexandrian, Panayiotis Mavroidis, Emma Sargent, Michael Allen, Christopher W. Schneider, Sotiri Stathakis

**Affiliations:** ^1^ Physics Department Louisiana State University Baton Rouge Louisiana USA; ^2^ Mary Bird Perkins Cancer Center Baton Rouge Louisiana USA; ^3^ Department of Radiation Oncology University of North Carolina Chapel Hill North Carolina USA; ^4^ CARTS Louisiana State University Baton Rouge Louisiana USA

**Keywords:** adaptive radiotherapy, CBCT, dashboard, DVH, NTCP, TCP

## Abstract

**Purpose:**

Adaptive radiotherapy (ART) is limited by the absence of reliable thresholds to trigger necessary plan adaptation. This work develops and verifies a web‐based, database‐backed dashboard that unifies geometric, dosimetric, and radiobiological metrics to support day‐to‐day ART decisions.

**Methods:**

Ten retrospectively selected patients were analyzed (Head and Neck (H&N), *n *= 5, 30–35 fx; prostate MR‐linac, *n *= 5, 5 fx). Daily dose was recalculated on CBCT‐derived pseudo‐CTs (pCT) (H&N) or on daily MR images (prostate). A Python/Dash dashboard with a PostgreSQL backend takes in RTStruct/RTDose, then performs structure name harmonization, computes per‐fraction DVHs, and derives five radar‐chart metrics: organ central point displacement, interfraction Dice (reported as 1–Dice), intrafraction Dice (OAR–PTV overlap vs reference), objective score (deviation from plan objectives), and a radiobiology score using TCP/NTCP to calculate *PI, PB*, and *P*+. Residuals, defined as the difference between the reference metric (MIM) and the corresponding dashboard calculated value (MIM—Dashboard), were calculated to assess agreement. Calculations were verified against MIM Maestro using residual plots, paired *t*‐tests (*α *= 0.05), and effect sizes.

**Results:**

Central point residuals were negligible (mean *<* 0.05 mm on all axes; max 0.45 mm). Interfraction Dice mean absolute difference was 0.08 (max 0.83); intrafraction Dice differences were smaller (overall mean residual −0.01). For score card dose endpoints, the largest mean difference was at D99.9% (1.31 Gy; 2.08%—below TG‐114′s 5% action level). Percent‐volume endpoints showed small residuals (overall mean +0.32%); absolute‐volume endpoints were near zero (overall mean +0.05 cm^3^). Radiobiology residuals were modest (mean: *PI* +1.78%, *P*+ −1.11%, *PB* +0.86%) with occasional outliers (max *PI* 8.69%). Three metrics reached statistical significance (central point, intra‐/inter‐fraction Dice), but effect sizes were negligible to small.

**Conclusions:**

The dashboard reproduces geometric, DVH, objective, and radiobiology metrics within acceptable limits relative to MIM, providing a credible foundation for ART decision support. Limitations include the projected DVH being a visualization (not dose accumulation) and radiobiology sensitivity to DVH sampling in steep gradients.

## 1 INTRODUCTION

Radiation therapy is traditionally delivered using a static treatment plan based on a CT scan acquired days to weeks before the first fraction. This assumes that patient anatomy remains stable throughout treatment. In practice, however, patients undergo substantial anatomical changes, including tumor shrinkage or growth, weight gain, weight loss, and variable organ filling, which can compromise target coverage and increase dose to organs‐at‐risk (OARs).^1^ To mitigate these uncertainties, population‐based margins are applied to the clinical target volume (CTV) and expanded into a planning target volume (PTV).^2^, ^3^ While effective for maintaining coverage, these margins increase irradiated volume and toxicity risk.

Adaptive radiotherapy (ART) aims to reduce reliance on large margins by monitoring anatomical and dosimetric changes and adjusting treatment accordingly.^4^ However, ART is not yet routine clinical practice due to technical and workflow barriers. ART has been used for multiple sites, but in this paper we will be focusing on two sites: Head and Neck (H&N) and prostate as they offer unique perspectives. Offline ART, where a new plan is generated after several fractions, is typically reserved for H&N patients, who experience progressive anatomical shrinkage during treatment.^1^, ^5^ Online ART, where plans are updated immediately before delivery, is increasingly feasible for prostate cancer patients, whose targets are subject to daily organ motion.^1^, ^6^ Despite these advances, there is no consensus on how or when to trigger adaptation.

Although dose‐volume histograms (DVHs) and isodose distributions are the cornerstones of plan evaluation, they primarily quantify the physical dose distribution, failing to fully capture the complex radiobiological consequences that determine clinical success.^2^ Radiobiological metrics such as tumor control probability (TCP), normal tissue complication probability (NTCP), and complication‐free TCP (*P*_+_) provide more clinically meaningful endpoints,^7^ but are rarely integrated into ART workflows.

Several gaps remain:

1. The absence of standardized, quantitative criteria for initiating ART.
2. A significant opportunity to enhance adaptation decisions through the greater integration of radiobiology‐based metrics.

Addressing these limitations would support more evidence‐based adaptation decisions and improve treatment personalization.

This work develops and evaluates a framework to address these limitations by providing ART decision support. The framework consists of three aims: (1) a database‐driven dashboard for tracking daily dose, DVHs, and adaptive triggers; (2) automated processing of patient imaging and dose data to evaluate workflow feasibility; and (3) incorporation of geometric, dosimetric, and radiobiological metrics to quantify adaptation need. The framework is tested in two disease‐site cohorts‐ H&N, representing progressive anatomical changes, and prostate patients, representing daily motion—to evaluate generalizability across adaptation contexts.

## 2 METHODS

### 2.1 Patient data and inclusion criteria

We retrospectively analyzed data from ten patients, evenly divided between two disease sites: H&N (*n* = 5) and prostate (*n* = 5).

**H&N**: The five H&N patients received conventionally fractionated definitive radiation therapy with a total dose prescription of approximately 70 Gy delivered over 30–35 fractions. Patients were treated with a conventional C‐arm Linac, Elekta Infintiy, VersaHD, and Harmony. Selection prioritized cases with daily setup imaging using cone‐beam CT (CBCT). Key structures evaluated included: *[Brachial Plexus Left and Right, Brainstem, Larynx, Mandible, Oral Cavity, Parotid Left and Right, Spinal Cord, Target Volumes]*.

**Prostate**: The prostate cohort consisted of five patients treated on an MR‐Linac (Elekta Unity) system with an ultrahypofractionated regimen of 36.25 Gy in 5 fractions. Key structures evaluated included: *[Bladder, Colon Sigmoid, Femur Left and Right, Penile Bulb, Rectum, Sacrum, Target Volume, Urethra]*.

For both cohorts, inclusion required the availability of the reference plan, dose, and structure set DICOM files. All data were anonymized and stored within a secure PostgreSQL database accessed by the dashboard backend.

### 2.2 Daily dose generation

Daily delivered dose distributions were generated separately for the H&N and prostate cohorts using site‐specific imaging workflows.

**H&N**: Daily CBCTs acquired prior to treatment were processed using the TheraPanacea AdaptBox software.^8^ Each CBCT was first rigidly registered to the planning CT in MIM. The aligned CBCT was then processed through the AdaptBox module to generate an AI‐based pseudo‐CT (pCT) with assigned Hounsfield units suitable for dose recalculation. Following pCT generation, structures were auto‐contoured using TheraPanacea's auto‐contouring function. These autocontours were reviewed and used as provided by the software and were not routinely hand‐edited prior to dose recalculation and dashboard import. The reference treatment plan was recalculated on each pCT to obtain the corresponding daily dose distribution. For analysis, five weekly fractions per patient (out of 30–35 total) were selected to sample both early and late treatment phases, reflecting anatomical change across the course.

**Prostate**: Each daily MRI was first rigidly aligned in Monaco to the reference CT to ensure geometric consistency across fractions. Using Monaco, the reference plan was transferred and recalculated on each daily MRI to represent delivered dose under a static‐plan workflow. This recalculation was performed using a bulk density override, in which each structure on the daily MRI was assigned the mean density of its corresponding structure on the reference CT. Because these patients were treated with an adaptive workflow, the daily structures generated during clinical treatment were already available and were used directly in this study. Five fractions per patient were analyzed to capture variability in target motion and organ filling across the short treatment course.

All daily dose distributions were exported in DICOM RT Dose format and imported into the dashboard database for downstream analysis.

### 2.3 Dashboard workflow

**FIGURE 1:**
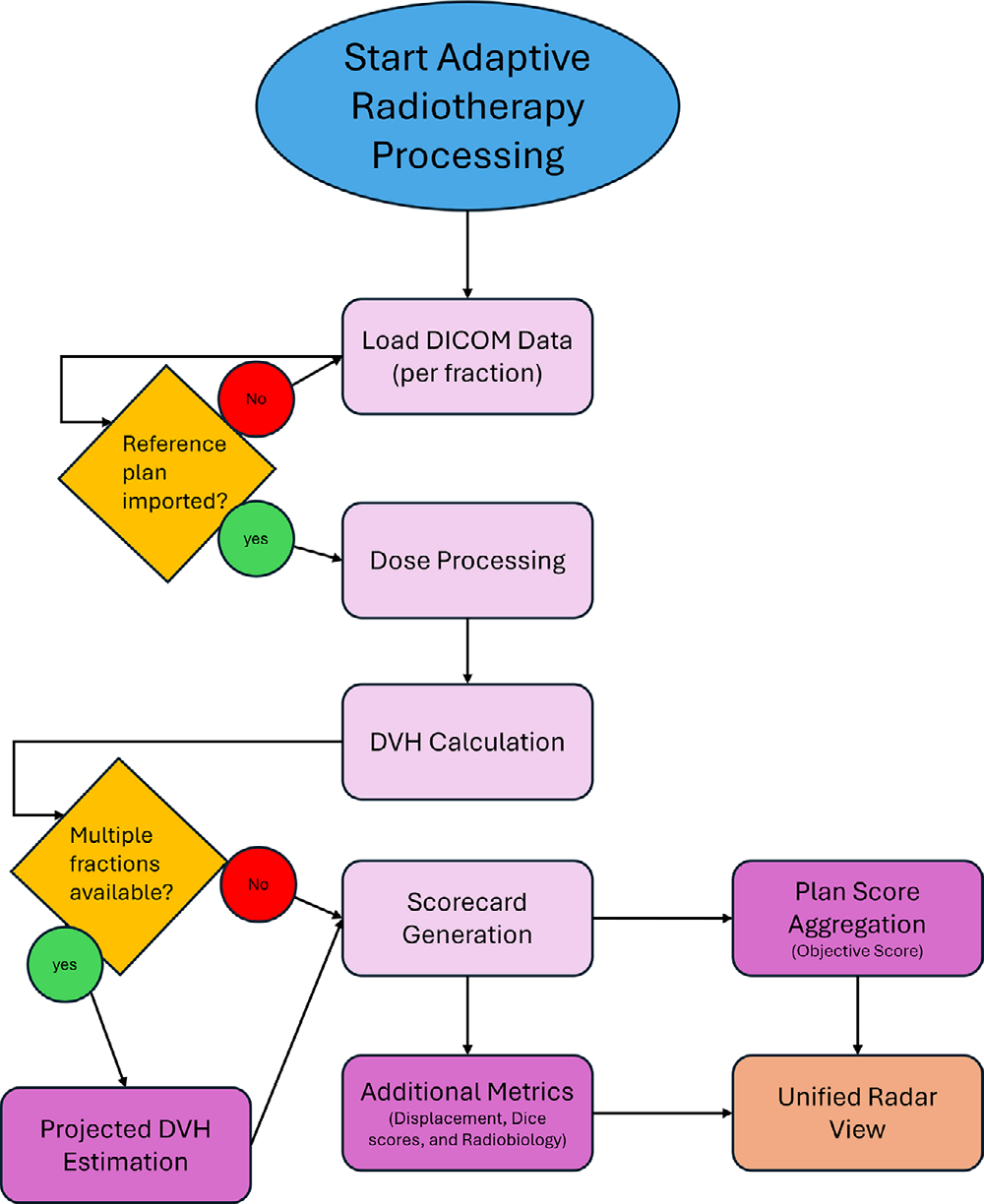
Automated dashboard workflow for adaptive radiotherapy. The pipeline processes DICOM data into dose distributions, DVHs, and scorecards, with results stored in PostgreSQL tables for real‐time visualization.

The dashboard automates ART data processing through a modular pipeline (Figure 1). DICOM data for each fraction (RTStruct, RTDose, and optionally RTPlan) are imported, dose grids are resampled and aligned to the structure set, and DVHs are computed for each contoured structure. Each stage of the pipeline writes results to PostgreSQL tables for subsequent visualization and decision support.

### 2.4 Structure set alignment and mapping

A key requirement for accurate dashboard evaluation is ensuring that anatomical structures are both geometrically aligned and consistently named across all fractions. This process combines spatial alignment of the structure sets with user‐guided name harmonization.

**Spatial alignment**: To enable longitudinal geometric analysis, all daily CBCT/MRI images are rigidly aligned to the patient's reference CT so that subsequent geometric calculations are carried out in the reference CT coordinate system. This common spatial frame ensures that metrics such as interfraction Dice similarity coefficients reflect anatomical change rather than image positioning,^9^ and it underpins accurate pCT generation, since the AdaptBox workflow assumes a rigidly aligned CBCT when creating a geometry‐consistent image for dose recalculation.^10^

### 2.5 Metric calculations

To quantify anatomical and dosimetric variation across treatment fractions, five core metrics were computed for each structure and displayed in a radar plot for visual comparison against the reference plan. Together, these metrics capture complementary aspects of daily treatment consistency. Although MIM does not support rapid per‐fraction evaluation or adaptive decision workflows, it computes the geometric and dose–volume endpoints (e.g., central point positions, DVH values) needed to verify the dashboard's intermediate calculations. A detailed description of this verification is provided in Section 2.7.

**FIGURE 2:**
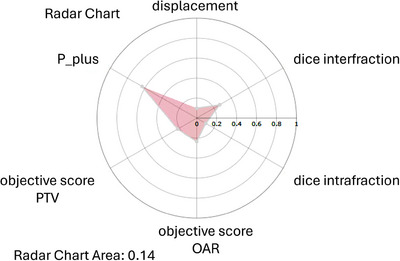
Radar chart for a H&N patient at fraction 7. The dashboard displays radar plots as a visual representation of the calculated metrics defined in Section 2.5, where the overall chart area serves as a visual cue for determining whether adaptation may be necessary.

Each of these five metrics result in a score from 0 to 1, where 0 represents perfect agreement with the reference plan and 1 denotes the maximum observed deviation across fractions. The values are simultaneously visualized on a pentagonal radar chart, allowing clinicians to assess deviations across multiple domains of treatment quality at a glance. The area enclosed by the radar polygon serves as a compact indicator of overall treatment deviation, with larger areas corresponding to greater deviations from the planned reference (Figure 2).

**Limitations of radar interpretation**: Adaptive triggers or action thresholds are not defined in this study, as the limited cohort size precludes establishing statistically robust criteria. Future work using larger patient cohorts may support the development of clinically meaningful trigger levels—potentially metric‐specific or site‐specific—depending on observed variability patterns and clinical priorities, as discussed in Section 4.1.6.

**Displacement score**: The displacement score quantifies the positional shift of an anatomical structure by comparing the location of its central point in the current fraction with that of the reference. This metric reflects interfraction motion and anatomical variability, which are critical in determining whether daily anatomy departs from the planned geometry. Registration for this calculation is described in Section 2.4; after registration, all central point coordinates are expressed in the reference CT coordinate system.

Displacement is calculated using the Euclidean distance between central points, normalized by the magnitude of the reference central point coordinates:

(1) $$Displacement=\frac{\sqrt{{\left(x_{in}-x_{i0}\right)}^{2}+{\left(y_{in}-y_{i0}\right)}^{2}+{\left(z_{in}-z_{i0}\right)}^{2}}}{\sqrt{x_{i0}^{2}+y_{i0}^{2}+z_{i0}^{2}}},$$

where (*x_i_*_0_ *, y_i_*_0_ *, z_i_*_0_) are the axes for the central point of structure *i* in the reference and (*x_in_, y_in_, z_in_*) are the axes of the central point of the same structure in the *n* ^th^ fraction. This scaling ensures comparability across structures of different sizes and locations. Because the scaled displacement can exceed 1 for large geometric shifts, values greater than 1 are explicitly capped at 1 to maintain a consistent 0–1 range across all radar metrics. Because the displacement score is sensitive to small positional shifts, it is particularly relevant in regions with steep dose gradients near critical OARs. A score near zero indicates minimal displacement from the reference, whereas larger values suggest substantial deviation and may support adaptive intervention when combined with other metric changes.

**Dice intrafraction and interfraction scores**: Dice similarity coefficients are used to measure spatial overlap between anatomical structures. In the context of ART, two types of Dice Scores are evaluated: the **intrafraction** score and the **interfraction** score. Both quantify spatial overlap, but they are applied differently in this work: within the same fraction (OAR vs. target) or between fractions (reference vs. daily).

**Intrafraction dice score**: The intrafraction score compares the overlap between a critical OAR and the high‐dose target volume (e.g., PTV) within the same fraction. It quantifies the degree of encroachment or separation between these two structures and is computed using:

(2) $$Dic\;e_{intra}=\frac{2\left|A_{OAR_{n}}\cap A_{PTV_{n}}\right|}{\left|A_{OAR_{n}}\right|+\left|A_{PTV_{n}}\right|}$$

where *|A*_OAR_ *|* is the absolute volume for the OAR in fraction *n*, where *n* is the current fraction number. *|A*_PTV_ *|* is the absolute volume for the PTV in fraction *n*.

High values indicate close spatial proximity or overlap, which may be undesirable if the OAR is receiving unintended dose. If overlap occurs in the reference for the PTVs and the OAR then the starting value is larger than zero. The radar chart represents the change from the reference plan, so the Dice Score intrafraction represented in the radar chart is computed using:

(3) $$\mathrm{Dic}e_{\mathrm{intra}\;\mathrm{radar}}=\left|\mathrm{Dic}e_{\mathrm{intra}\;\mathrm{reference}}-\mathrm{Dic}e_{\mathrm{intra}\;n}\right|$$

Changes in this score over time can indicate worsening geometry due to anatomical shifts.

**Interfraction dice score**: This score evaluates how much a given structure changes shape and position from one fraction to another relative to the reference. The Dice coefficient is defined as:

(4) $$\mathrm{Dic}e_{\mathrm{inter}}=\frac{\left|2\cdot |A_{0}\cap A_{n}|\right|}{\left|A_{0}\right|\;\left|A_{n}\right|},$$

where *A*_0_ is the binary mask of the structure from the reference, and *A_n_* is the same structure in the *n* ^th^ fraction. A Dice Score of 1 indicates perfect agreement, while a score closer to 0 implies poor spatial overlap. Large interfraction variation may suggest that replanning is necessary to maintain accurate targeting and OAR sparing.

Because higher Dice values represent better agreement, but the radar visualization is designed such that larger areas correspond to worse performance, the dashboard inverts this metric by computing (1 − Dice_inter_). This transformation ensures consistency across all radar scores, where higher values uniformly indicate worse geometric, dosimetric, or biological outcomes.

Both Dice Scores provide structural insights beyond DVHs, enabling clinicians to better assess the need for adaptive strategies based on geometric considerations.

**Objective score**: The objective score quantifies how well each anatomical structure meets its dose‐volume constraints relative to the reference (typically the original planned dose in the reference). This metric provides a standardized way to flag when a structure's dosimetric behavior deviates meaningfully from the original treatment intent.

For each structure, the deviation is calculated based on a user‐defined set of dose‐volume objectives (e.g., *D*_99_ for PTVs, *V*_30_ for OARs). The deviation is normalized relative to the reference value using the following formula:

(5) $$Objective_{s}core=\sum_{i=1}^{n}\sqrt{{\left(\frac{D_{ij}-d_{i0}}{x}\right)}^{2}+{\left(\frac{V_{ij}-v_{i0}}{100}\right)}^{2}},$$

where *D_ij_* is the objective dose received by structure *i* in fraction *j*, *d_i_*_0_ is the corresponding objective dose in the reference, *x* is the prescription dose, *V_ij_* is the objective volume received by structure *i* in fraction *j*, and *v_i_*_0_ is the corresponding objective volume in the reference plan.^11^ Each objective yields a deviation value, and the objective scores for a given fraction are averaged to produce a single score. Because the goal is to closely replicate the reference's objectives, values nearer to zero indicate greater similarity between the current and reference plans. When displaying the radar (Figure 2), the composite objective score is split into two components: a sum over OAR objectives and a sum over PTV objectives. Because there are typically more OAR objectives and each objective is weighted equally, a single combined score can be dominated by the OARs. Reporting the two components separately helps the user interpret the plan's effect on PTVs and OARs independently.

Because the objective score is a composite value derived from dose–volume endpoints, it cannot be directly compared with MIM as a standalone metric; MIM does not compute an analogous score. Instead, validation was performed at the level of the score card dose–volume results—the sole inputs to the objective score formula. Consistent dose and volume endpoints between the dashboard and MIM ensure that any difference in the objective score would arise only from those underlying inputs.

**Radiobiology score (*P_B_*, *P_I_, P*_+_)**: Radiobiological modeling was incorporated to evaluate the therapeutic balance between tumor control and normal‐tissue injury. TCP and NTCP were computed using standard Poisson‐based TCP formulations and the Lyman–Kutcher–Burman (LKB) NTCP model, following established methods and parameterizations in the literature^7^, ^12^, ^13^. These models were applied directly to each structure's DVH to generate the composite metrics:

(6) $$P_{B}={\prod_{i=1}^{m}TCP}_{i}$$

(7) $$P_{I=}1-\;\prod_{j=1}^{n}\left(1-NTCP_{j}\right)$$

(8) $$P_{+}=P_{B}\left(1-P_{I}\right),$$

where *P_B_* is the probability that all targets are controlled, *P_I_* the probability that at least one OAR incurs injury, and *P*_+_ the probability of complication‐free tumor control.

**Radar radiobiology score**: To align with the radar chart convention where larger values represent greater deviation from the reference plan, daily radiobiology is summarized by the absolute change in *P*_+_ relative to the reference fraction:

(9) $$\mathrm{Scor}e_{\mathrm{radio}}=\left|{P_{+}}_{0}-{P_{+}}_{i}\right|$$

High therapeutic quality (high *P*_+_) therefore yields small scores, whereas reductions in tumor control or increases in complication risk elevate the score. This normalized value is displayed alongside the other metrics to provide a balanced representation of daily treatment quality.

**Validation**: Unlike the other metrics, MIM does not calculate the specific radiobiological indices used in this study (P_+_, *P_B_*, and *P_I_*). However, these metrics depend solely on dose–volume histogram (DVH) data, which are available from both systems and serve as the complete set of inputs to the radiobiological formulas. To accomplish this, the same dose and structure DICOMs were imported into both MIM and the dashboard so that each system generated its own DVHs. The MIM‐derived DVHs were then exported and processed externally (in an Excel‐based script) using the same formulas ((6)–(9)) implemented in the dashboard, while the dashboard applied those formulas directly to its internally computed DVHs. Any differences in the resulting radiobiology scores therefore reflect only differences in the DVH generation process, isolating the reliability of the dashboard's radiobiology implementation.

### 2.6 Dashboard design and PostgreSQL integration

The core of this ART platform is a web‐based dashboard that provides a unified interface for monitoring daily dose metrics, anatomical changes, and potential adaptation needs. Built in Python using the Dash framework, the dashboard is supported by a PostgreSQL backend that persistently stores patient metadata, mapped structure names, per‐fraction and cumulative scores, radiobiological metrics, dose–volume histograms (DVHs), and historical dose records across fractions.

The dashboard incorporates several visualization and evaluation modules to support adaptive decision‐making.

For dashboard interface examples and detailed descriptions see the Supplemental document Figures S1 and S2.

**FIGURE 3:**
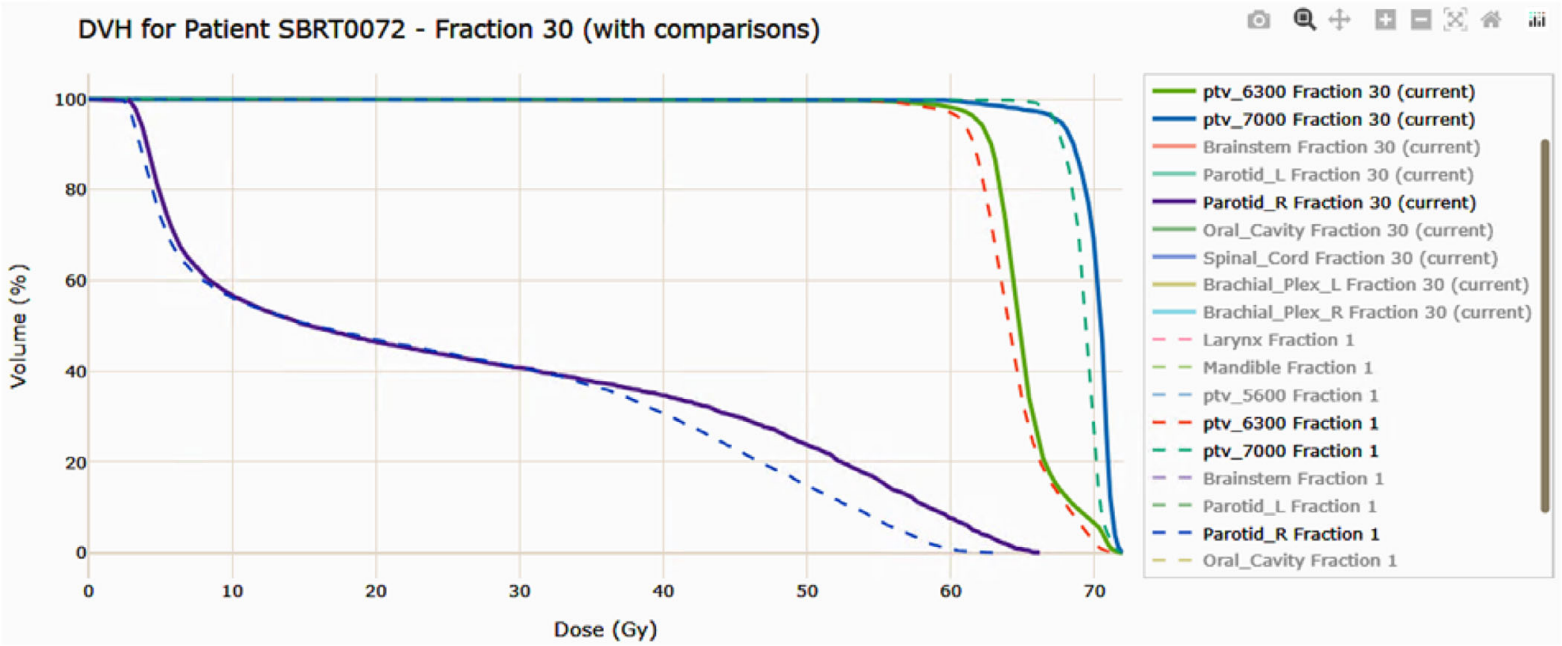
DVH per fraction is available when viewing the overall fraction score diagrams. The dashboard lets users compare the current fraction with any previous fractions. Example H&N: PTV 6300, PTV 7000, and parotid right for fractions 1 vs 30.

**Dose volume histogram**: The dose–volume histogram (DVH) and dose viewer enables users to compare the current with any previous fractions for any selected structure while simultaneously overlaying dose distributions on CT slices. This functionality enables assessment of dosimetric variation between fractions and relative to the original plan. An example is provided in Figure 3, which illustrates a H&N case with PTV 6300, PTV 7000, and the right Parotid Gland compared between fractions 1 and 30.

**Projected DVHs (structure‐specific)**: In addition to per‐fraction DVHs, the dashboard generates a projected DVH for each structure to summarize the average dose‐volume behavior observed over the delivered fractions. For a given structure i and k delivered fractions, let Hi,f (db) denote the DVH ordinate (volume or percentage) in dose bin db for fraction f. To ensure a consistent dose grid, the dose bins from the first available fraction for that structure are used as a reference, and DVHs from the remaining fractions are linearly interpolated onto that grid. The projected DVH is then computed as a binwise arithmetic mean:

(10) $$\;H_{i,proj}^{\left(k\right)}\left(d_{b}\right)=\frac{1}{k}\sum_{f=1}^{k}H_{i,f}\left(d_{b}\right).$$

**FIGURE 4:**
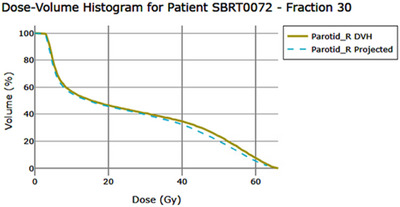
Fraction 30 DVH is compared to the projected DVH. The projected DVH is the binwise mean of the delivered per‐fraction DVHs on a common dose grid. Because each per‐fraction DVH is derived from a full‐dose recalculated RTDose, the projected curve spans the full prescription dose range even when only a subset of fractions is available.

By construction, the projected DVH remains bounded between 0–100% because the ordinate represents normalized volume and each per‐fraction DVH is derived from a daily RTDose recalculated on that day's anatomy using the full prescription dose distribution; therefore, the projected DVH is displayed over the full dose range even when only a subset of fractions has been delivered. No additional scaling is applied during the projection step itself. An example of this visualization is shown in Figure 4. This visualization is intended to show how the average DVH pattern would appear if the observed behavior continued; it is not a voxelwise dose accumulation.

**Limitations of the projected DVH**. Because projected DVHs average per‐fraction DVHs rather than accumulating voxelwise dose, they do not preserve spatial correspondence and must not be interpreted as accumulated dose. The method is also sensitive to registration quality, contour edits, imaging uncertainties, and the assumption that delivered fractions are representative of the remaining course. Additional technical details and discussion of these limitations are provided in the Supplementary Material.

**Scope of use**. Here, the projected DVH is used strictly as a DVH‐averaging visualization across delivered fractions; we make no claim that it represents the true accumulated dose or a validated metric, and any formal validation is outside the scope of this work.

**Radar chart**: To provide an integrated view of geometric and dosimetric consistency, the dashboard generates fraction‐specific radar plots. These visualizations summarize the metrics defined in Section 2.5, consolidating geometric displacement, dosimetric variation, and biological variation into a single chart. The enclosed area provides an intuitive representation of overall deviation across metrics. Figure 2 demonstrates this functionality for a H&N patient at fraction 2, highlighting its role in guiding adaptation decisions.

**FIGURE 5:**
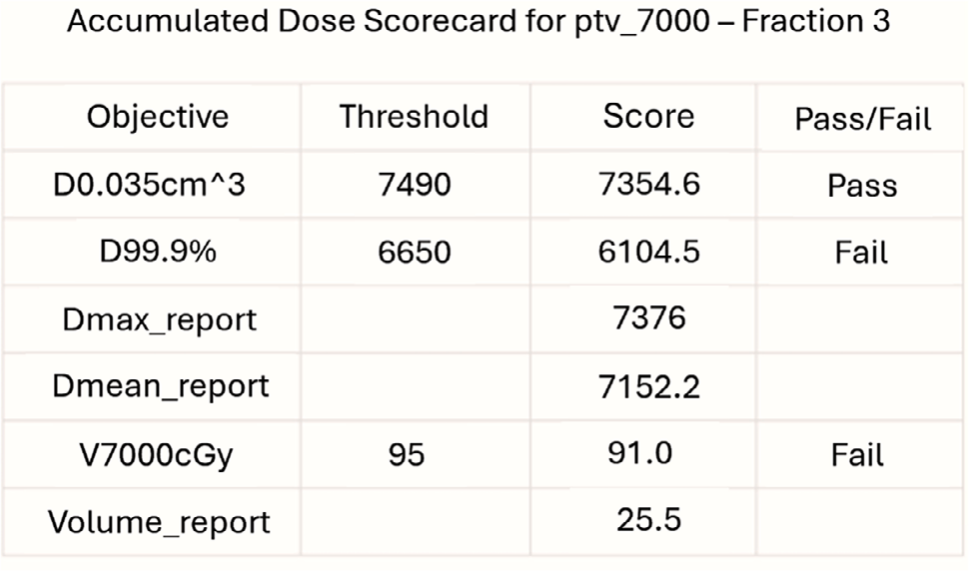
Example scorecard for PTV 7000 at the third treatment fraction. Using template defined in Table 1.

**Score card**: For detailed per‐fraction evaluation, scorecards are automatically generated for each mapped structure. These scorecards follow H&N and prostate templates from the Monaco Treatment Planning System (TPS) (Table 1 and Table 2), ensuring alignment with established clinical benchmarks. Figure 5 illustrates an example for PTV 7000 at the third treatment fraction.

**TABLE 1:** H&N score card clinical objectives.

Structure	Objective	Rule	Threshold	Tolerance (±)	Template
Brachial Plex L	D0.035cm3	*≤*	6600 cGy	500 cGy	≤ 6600 cGy (500)
Brachial Plex R	D0.035cm3	*≤*	6600 cGy	500 cGy	*≤* 6600 cGy (500)
Brainstem	V5400cGy	*<*	0.035 cm3	0 cm3	*<* 0.035 cm3 (0)
Larynx	V7350cGy	*≤*	0.035 cm3	0 cm3	*≤* 0.035 cm3 (0)
Larynx	Dmean	*≤*	4000 cGy	0 cGy	*≤* 4000 cGy (0)
Mandible	D0.035cm3	*≤*	7100 cGy	100 cGy	*≤* 7100 cGy (100)
Oral Cavity	Dmean	*≤*	3000 cGy	0 cGy	*≤* 3000 cGy (0)
Parotid L	V3000 cGy	*≤*	50%	0%	*≤* 50% (0%)
Parotid L	Dmean	*≤*	2600 cGy	0 cGy	*≤* 2600 cGy (0)
Parotid R	V3000 cGy	*≤*	50%	0%	*≤* 50% (0%)
Parotid R	Dmean	*≤*	2600 cGy	0 cGy	*≤* 2600 cGy (0)
Spinal Cord	V4500cGy	*≤*	0.035 cm3	0 cm3	*≤* 0.035 cm3 (0)
PTV 5412	V5412cGy	*≥*	95%	0%	*≥* 95% (0%)
PTV 5412	D99.9%	*≥*	5000 cGy	0 cGy	*≥* 5000 cGy (0)
PTV 5600	V5600cGy	*≥*	95%	0%	*≥* 95% (0%)
PTV 5600	D99.9%	*≥*	5320 cGy	0 cGy	*≥* 5320 cGy (0)
PTV 5940	V5940cGy	*≥*	95%	0%	*≥* 95% (0%)
PTV 5940	D99.9%	*≥*	5600 cGy	0 cGy	*≥* 5600 cGy (0)
PTV 6300	V6300cGy	*≥*	95%	0%	*≥* 95% (0%)
PTV 6300	D99.9%	*≥*	5985 cGy	0 cGy	*≥* 5985 cGy (0)
PTV 6600	V6600cGy	*≥*	95%	0%	*≥* 95% (0%)
PTV 6600	D99.9%	*≥*	6300 cGy	0 cGy	*≥* 6300 cGy (0)
PTV 6996	D0.035cm3	*≤*	7490 cGy	210 cGy	*≤* 7490 cGy (210)
PTV 6996	V6996cGy	*≥*	95%	0%	*≥* 95% (0%)
PTV 6996	D99.9%	*≥*	6650 cGy	0 cGy	*≥* 6650 cGy (0)
PTV 7000	D0.035cm3	*≤*	7490 cGy	210 cGy	*≤* 7490 cGy (210)
PTV 7000	V7000cGy	*≥*	95%	0%	*≥* 95% (0%)
PTV 7000	D99.9%	*≥*	6650 cGy	0 cGy	*≥* 6650 cGy (0)

Clinical objectives used for the H&N scorecards. “Tolerance (±)” means the listed amount is applied symmetrically about the threshold. A value of 0 indicates no additional tolerance.

**TABLE 2:** Prostate score card clinical objectives.

Structure	Objective	Rule	Threshold	Tolerance (±)	Template
Bladder	V3700cGy	*≤*	10 cm3	10 cm3	*≤* 10 cm3 (10)
Bladder	V1810cGy	*≤*	40%	5%	*≤* 40% (5%)
Bowel	D0.035cm3	*≤*	3000 cGy	500 cGy	*≤* 3000 cGy (500)
Bowel	D1cm3	*≤*	2700 cGy	300 cGy	*≤* 2700 cGy (300)
Femur L	V1450cGy	*≤*	5%	5%	*≤* 5% (5%)
Femur L	D1cm3	*≤*	1990 cGy	0 cGy	*≤* 1990 cGy (0)
Femur R	V1450cGy	*≤*	5%	5%	*≤* 5% (5%)
Femur R	D1cm3	*≤*	1990 cGy	0 cGy	*≤* 1990 cGy (0)
Penile Bulb	Dmean	*≤*	2950 cGy	0 cGy	*≤* 2950 cGy (0)
Rectum	D0.5cm3	*≤*	3806 cGy	194 cGy	*≤* 3806 cGy (194)
Rectum	V3600cGy	*≤*	1 cm3	2 cm3	*≤* 1 cm3 (2)
Rectum	V3260cGy	*≤*	10%	5%	*≤* 10% (5%)
Rectum	V2900cGy	*≤*	20%	5%	*≤* 20% (5%)
Rectum	V1810cGy	*≤*	50%	5%	*≤* 50% (5%)
Sigmoid	D0.035cm3	*≤*	3000 cGy	150 cGy	*≤* 3000 cGy (150)
Urethra	D0.03cm3	*≤*	4200 cGy	150 cGy	*≤* 4200 cGy (150)
PTV 3625	D98%	*≥*	3625 cGy	185 cGy	*≥* 3625 cGy (185)
PTV 3625	D99%	*≥*	3440 cGy	70 cGy	*≥* 3440 cGy (70)

Clinical objectives used for the prostate scorecards. “Tolerance (±)” means the listed amount is applied symmetrically about the threshold. A value of 0 indicates no additional tolerance.

**FIGURE 6:**
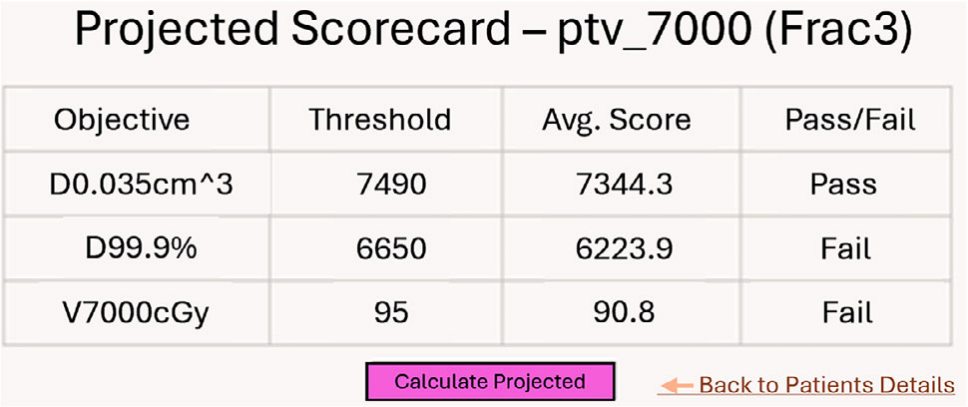
Projected scorecard for PTV 7000 at the third fraction, calculated by averaging DVHs from fractions 1 through 3. The magenta “Calculate projected” button in the dashboard generates this view. Using template defined in Table 1.

**Projected scorecard**: In addition to per‐fraction assessments, the dashboard generates projected scorecards that aggregate structure‐specific objectives across all fractions up to the current one. Each objective is reported as the mean of its per‐fraction values, while pass/fail status is determined using the same thresholds defined in the template scorecard. This cumulative view supports longitudinal evaluation of treatment performance. Figure 6 shows an example for PTV 7000 at fraction 3, computed by averaging DVHs from fractions 1 through 3.

Dashboard views retrieves and updates data from the PostgreSQL database, which stores patients, structures, DVHs, and derived metrics.

#### 2.6.1 Dose distribution visualization

The dashboard provides multiple visualization tools to support clinical interpretation and monitoring of anatomical and dosimetric changes throughout the treatment course.

**FIGURE 7:**
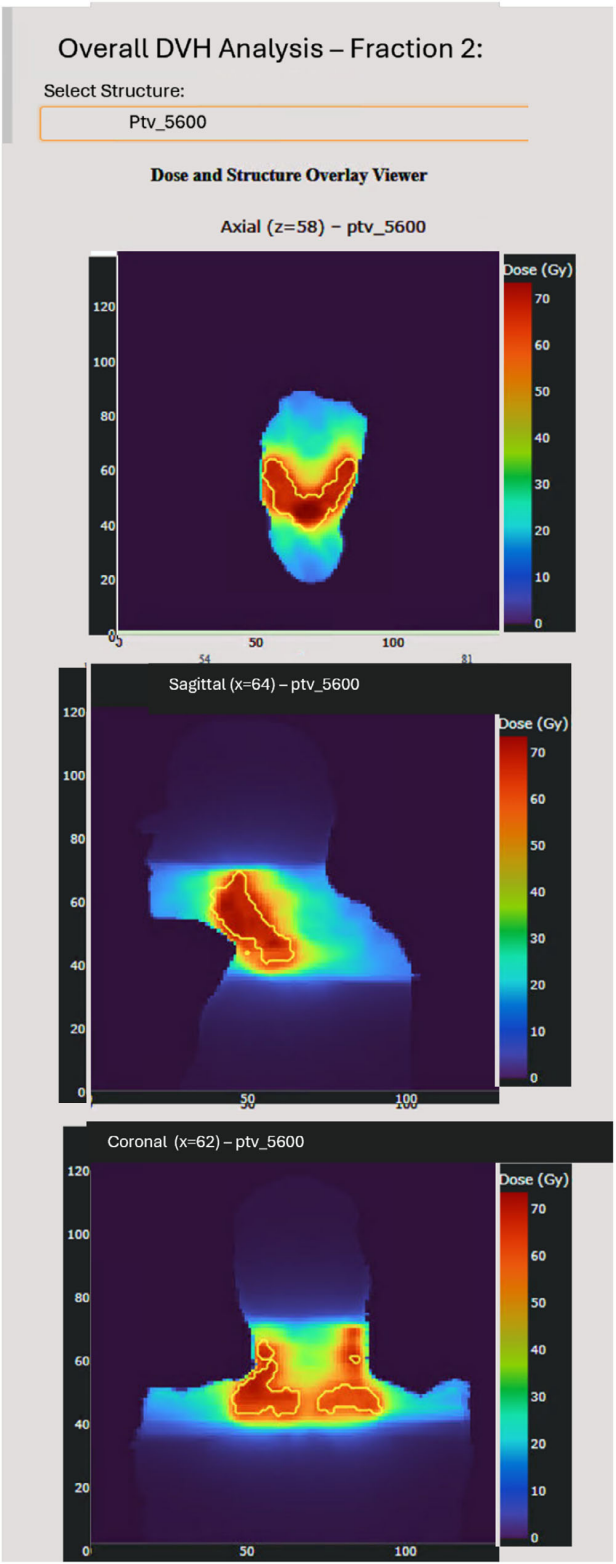
Orthogonal dose distributions (axial, sagittal, coronal) for a H&N case (fraction 2) with the PTV 5600 contour overlaid in its anatomical position. The color scale represents dose in Gy.

**Dose overlay viewer**: Orthogonal anatomical slices (axial, sagittal, coronal) display the dose distribution with user selected structure contours geometrically overlaid in their correct anatomical positions. This visualization allows users to inspect the spatial relationship between dose and anatomy, making it easy to evaluate the dose received within the contoured structure. Figure 7 shows a H&N case (fraction 2) with the PTV 5600 contour overlaid on the dose distribution. The color scale represents dose in Gy.

All displays are generated dynamically based on the selected patient, structure, and fraction, enabling real‐time exploration and facilitating clinical decision making regarding adaptive needs.

**FIGURE 8:**
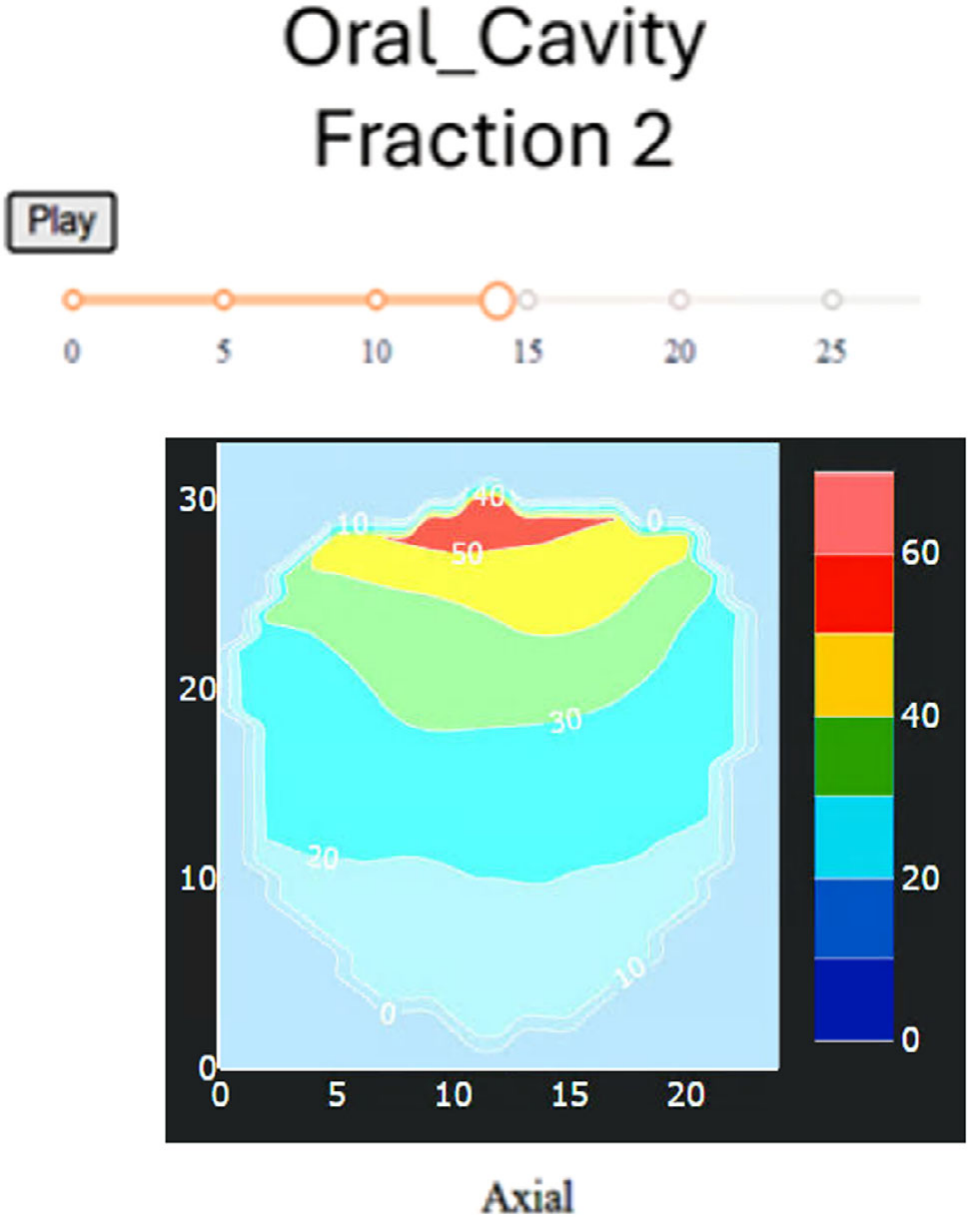
Dose overlay viewer for the oral cavity (fraction 2). The Play control animates the dose through all slices containing the structure, while the slider enables slice‐specific inspection. The color bar maps dose (Gy) to color.

**Dose‐per‐structure animation**: This view isolates the dose distribution within the boundaries of the selected structure. Only voxels inside the contour are displayed, allowing users to examine how dose is deposited across the structure. The display can be animated through all relevant slices using the Play button or inspected slice‐by‐slice with the slider. The color bar indicates dose in Gy. Figure 8 shows an example for the oral cavity at fraction 2.

### 2.7 Methods of verification

Verification focused on whether the dashboard provides reliable results when tracking metrics over time. Because inaccurate metric calculations would undermine any downstream clinical decisions, verification was performed at the metric level.

The same patients, dose distributions, and structure sets were imported into both the dashboard and MIM software. Each metric was calculated independently, and the paired differences between results (MIM − Dashboard) were computed. Residuals, defined as the difference between the reference metric (MIM) and the corresponding dashboard calculated value (MIM—Dashboard), were used to quantify the agreement between the two methods. Residual plots were then generated to visualize the distribution of these differences for each metric, with an ideal mean difference of zero indicating agreement between methods. The mean and maximum absolute differences were further assessed to quantify the magnitude of discrepancies.

To statistically assess reliability, paired *t*‐tests were performed on these paired differences under identical conditions—same patient, dose, and structure set—ensuring that the only variable was the calculation method.^14^ A significance level of *p < *0.05 was used to determine statistical differences. For metrics that showed a statistically significant difference, Cohen's *d* was additionally calculated as a standardized effect size to quantify the magnitude of those differences.^15^

## 3 Results

### 3.1 Results: Dashboard calculation verification

Employing the dashboard through the current adaptation workflow requires the calculation metrics to be verified. These values were verified by computing the residuals of MIM and the dashboard.

Residuals, defined in Section 2.7 (positive values meaning MIM *> *Dashboard), were calculated for each metric using the same patient, fraction, and structure inputs. Residual plots summarize these paired differences, with zero indicating perfect agreement.

#### 3.1.1 Central point verification

**FIGURE 9:**
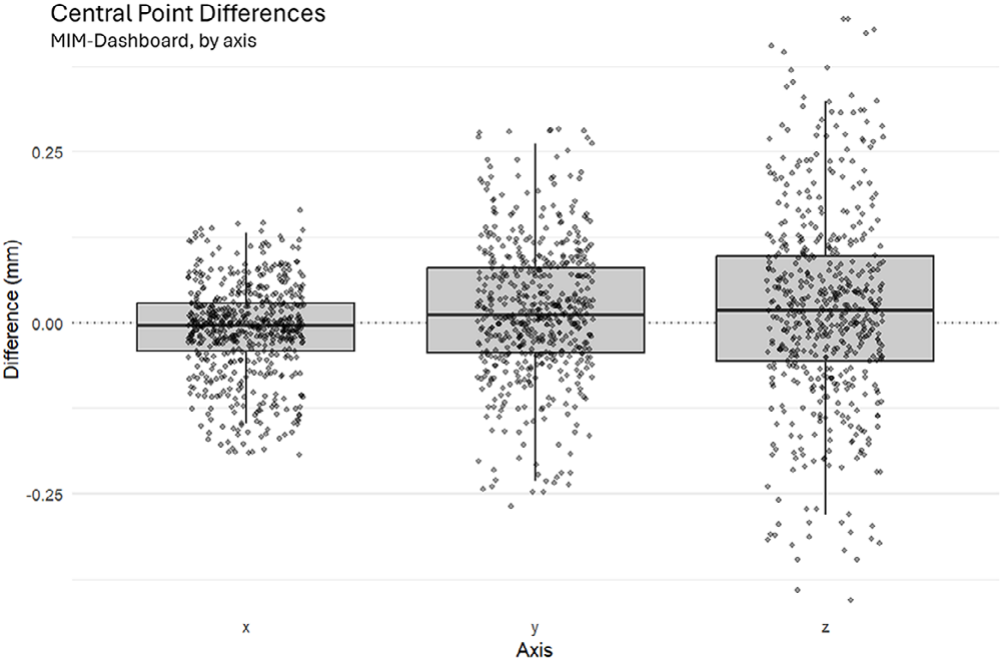
MIM software—dashboard calculations. The same central points calculated using MIM software and the dashboard. Shows small differences between the two calculations. The thick horizontal line within each box represents the median.

Central point (defined in Section 2.5) showed residuals per axis as summarized in Figure 9. Mean residuals were minimal: −0.01 mm (x), +0.02 mm (y), and +0.02 mm (z), with distributions tightly centered near zero across all patients and fractions.

#### 3.1.2 Dice score verification

**FIGURE 10:**
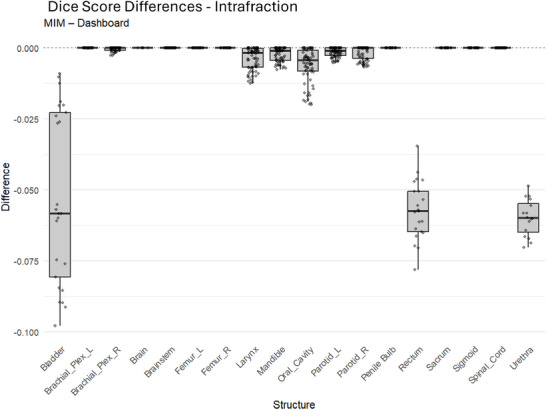
Intrafraction Dice Score comparison between MIM software and the dashboard. Differences are shown as MIM minus dashboard.

**Dice score intrafraction verification**: Figure 10 shows intrafraction residuals. The largest mean residual occurred in the bladder (−0.06), followed by the urethra (−0.06) and rectum (−0.03). Most remaining structures had residuals near zero. The overall mean intrafraction residual across all structures was −0.01.

**FIGURE 11:**
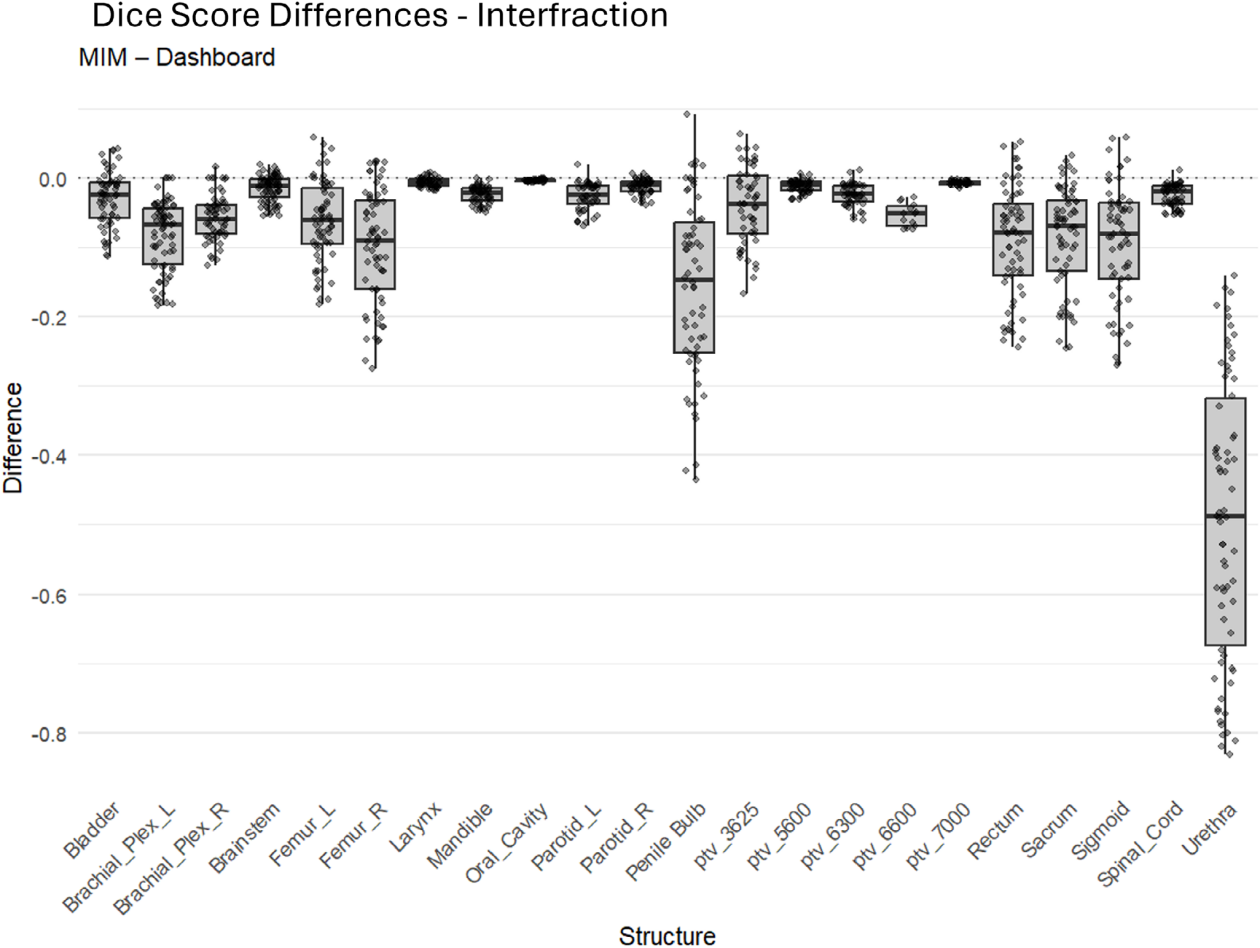
Interfraction dice score comparison between MIM software and the dashboard. Differences are shown as MIM minus dashboard.

**Dice score interfraction verification**: Figure 11 shows interfraction residuals. The urethra had the largest mean residual (−0.49). All structures showed negative mean residuals (range 0 to −0.49), indicating consistently higher Dice values from the dashboard. The overall mean interfraction residual was −0.08.

#### 3.1.3 Score card verification

**FIGURE 12:**
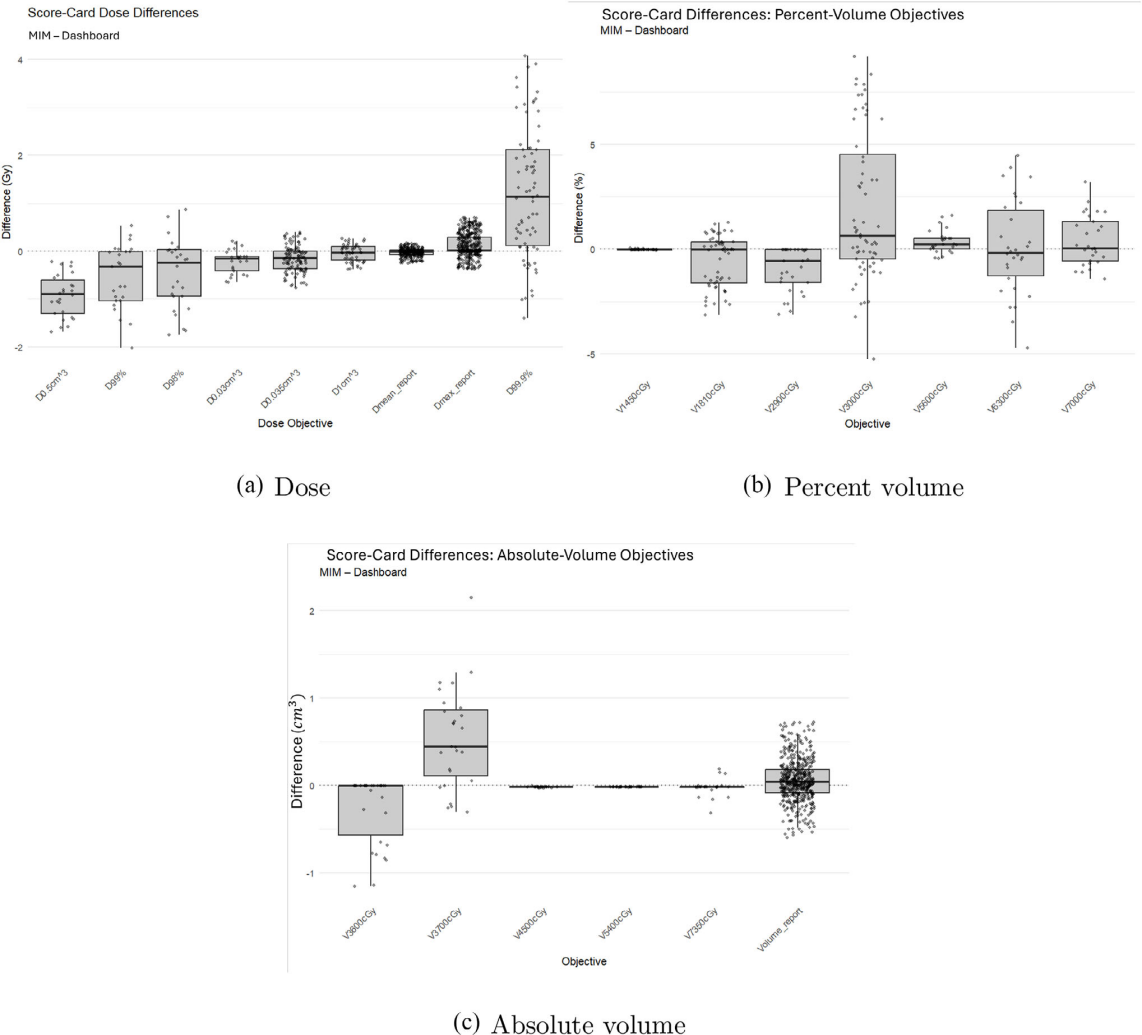
MIM software versus dashboard calculations. The same score card objectives were calculated using MIM software and the dashboard: (a) dose objectives (differences as large as 5.4 Gy with a mean difference of 0.25 Gy), (b) percent‐volume objectives, and (c) absolute‐volume objectives.

Figure 12A summarizes residuals for dose‐based score card objectives. The largest mean difference occurred at D99.9% (1.31 Gy). The overall mean residual across all dose objectives was 0.03 Gy.

Figure 12B shows residuals for percent‐volume metrics. The largest mean difference was V3000 cGy (+1.95%). Across all percent‐volume objectives, the overall mean residual was +0.32%.

Figure 12C summarizes residuals for absolute‐volume objectives. The largest mean difference was V3700cGy (+0.54 (cm3)). The overall mean residual across all absolute‐volume objectives was +0.05 (cm3).

#### 3.1.4 Radiobiology verification

**FIGURE 13:**
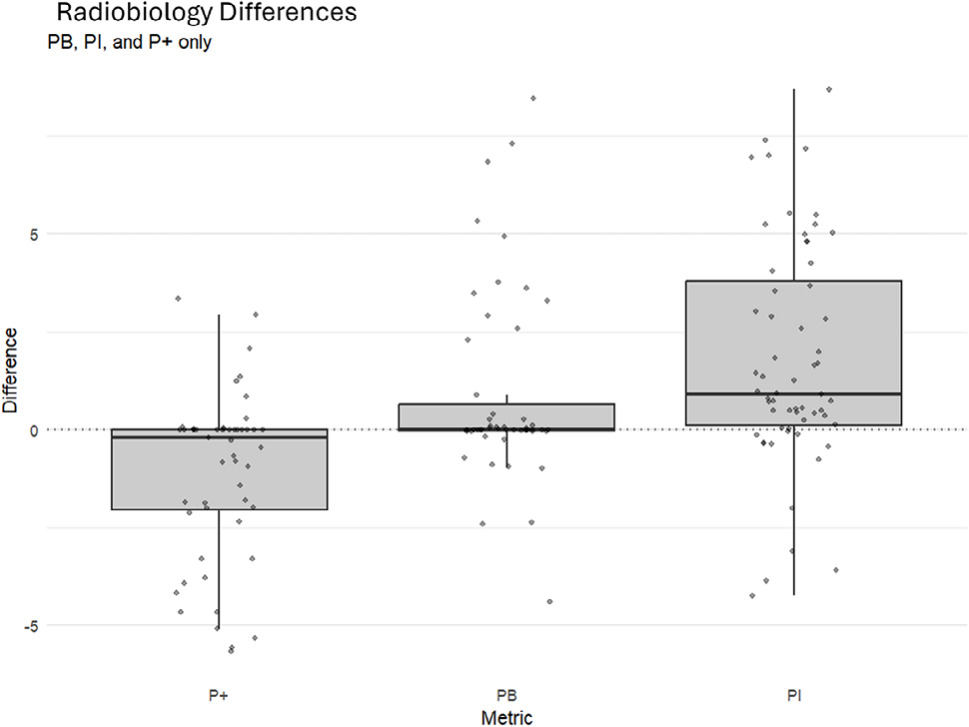
MIM software versus dashboard calculations. Radiobiological metrics calculated using MIM software and the dashboard.

Figure 13 shows residuals. Mean differences were: +1.78% for *P_I_*, −1.11% for *P*_+_, and +0.86% for *P_B_*.

#### 3.1.5 Validation statistics

Tables 3 and 4 summarize the mean and maximum absolute differences (*|*∆*|*) across all metrics. In general, mean *|*∆*|* values were small relative to the corresponding maxima, indicating that the largest discrepancies were isolated cases.

**TABLE 3:** Absolute differences for axes, dice, and dose metrics.

Metric (unit)	Mean *|*∆*|*	Max *|*∆*|*
*Axes*		
x (mm)	0.05	0.19
y (mm)	0.08	0.28
z (mm)	0.11	0.45
*Overlap / Segmentation*		
Dice — intrafraction (unitless)	0.01	0.13
Dice — interfraction (unitless)	0.08	0.83
*Dose objectives*		
D0.035 cm^3^ (Gy)	0.31	1.11
D0.03 cm^3^ (Gy)	0.33	1.00
D0.5 cm^3^ (Gy)	0.93	2.16
D1 cm^3^ (Gy)	0.17	0.64
D98% (Gy)	0.72	1.96
D99% (Gy)	0.73	2.10
D99.9% (Gy)	1.53	5.35
*D*max, report (Gy)	0.31	1.32
*D*mean, report (Gy)	0.18	0.77

Mean and maximum absolute differences (*|*∆*|*) for axes, Dice, and dose objectives. Tinted rows mark the largest Max *|* ∆ *|* within each section.

**TABLE 4:** Absolute differences for volume and radiobiology metrics.

Metric (unit)	Mean *|*∆*|*	Max *|*∆*|*
*Volume objectives (%)*		
V1450 cGy (%)	0.01	0.07
V1810 cGy (%)	1.01	3.13
V2900 cGy (%)	0.95	3.10
V3000 cGy (%)	2.87	9.18
V5600 cGy (%)	0.47	1.61
V6300 cGy (%)	1.74	4.72
V7000 cGy (%)	0.96	3.21
*Volume objectives* (cm^3^)		
V3600 cGy (cm^3^)	0.26	1.16
V3700 cGy (cm^3^)	0.62	2.15
V4500 cGy (cm^3^)	0.02	0.03
V5400 cGy (cm^3^)	0.02	0.02
V7350 cGy (cm^3^)	0.07	0.32
Volume report (cm^3^)	0.19	0.72
*Radiobiology*		
*P*_+_ (%)	1.59	5.66
*P_B_* (%)	1.38	8.46
*P_I_* (%)	2.43	8.69

Mean and maximum absolute differences (*|*∆*|*) for volume objectives and radiobiology metrics. Blue rows mark the largest Max *|* ∆ *|* within each metric.

**Central point**: The *z*‐axis exhibited the largest absolute maximum difference (0.45 mm); the absolute mean difference was 0.11 mm (Table 3). **Dice scores**: Interfraction Dice had the largest absolute maximum difference (0.83), with absolute mean difference 0.08 (Table 3). **Score‐card dose objectives**: The largest absolute maximum difference occurred at D99.9% (5.35 Gy); the absolute mean difference was 1.53 Gy (Table 3). **Score‐card volume objectives**: The largest absolute maximum difference occured at V3000 cGy (9.18%) with an absolute mean of 2.87%. V3700 cGy had the largest maximum absolute difference (2.15 (cm^3^)) and an absolute mean of 0.62 (cm^3^) (Table 4). **Radiobiology**: *P_I_* had the largest absolute maximum difference (8.69%), with absolute mean difference 2.43%; *P*_+_ and *P_B_* showed similar patterns (Table 4).

Table 5 summarizes paired *t*‐test results. Three metrics showed *p < *0.05: Central point (x), Dice interfraction, and Dice intrafraction.

**TABLE 5:** Statistical tests and 95% confidence intervals.

Metric	*n*	Mean diff	*t*‐stat	*p*‐value	95% CI (low)	95% CI (high)
Central point (x)	654	−0.02	−3.07	2.23 *×* 10−3	−0.04	−0.01
Central point (y)	654	0.01	0.36	0.72	−0.03	0.05
Central point (z)	654	0.04	1.51	0.13	−0.01	0.10
Dice—interfraction	1267	−0.08	−17.60	1.09 *×* 10−63	−0.08	−0.06
Dice—intrafraction	827	−0.01	−12.00	1.27 *×* 10−30	−0.01	−0.01
Score card—Dose (Gy)	1309	−0.21	−1.90	5.77 *×* 10−2	−0.42	0.01
Score card—Volume (%)	314	−0.30	−1.01	0.32	−0.88	0.28
Score card—Volume report	582	−0.27	−0.87	0.38	−0.88	0.34
Radiobiology—*P*_+_	62	−1.16	−1.78	8.04 *×* 10−2	−2.46	0.15

Summary statistics with *t*‐tests and 95% CIs. Cells in light green indicate *p < *0.05. Note that statistical significance need not imply practical importance.

For these metrics, standardized effect sizes (Cohen's *d*) were computed (Table 6) to quantify the magnitude of the paired differences.

**TABLE 6:** Effect size summary.

Metric	*n*	Mean diff	SD	Cohen's *d*	Hedges’ *g*	Wilcoxon *p*
Central point (x)	654	−0.02	0.19	−0.12	−0.12	7.88 *×* 10−4
Dice—interfraction	1688	−0.06	0.17	−0.43	−0.43	8.09 *×* 10−122
Dice—intrafraction	827	−0.01	0.02	−0.42	−0.42	5.86 *×* 10−59

Effect sizes for metrics with low *p*‐values. Color encodes magnitude: 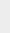 negligible *|d| < *0.2, 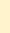 small 0.2–0.5, 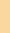 medium 0.5–0.8, 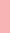 large *≥* 0.8.

## 4 DISCUSSION

### 4.1 Discussion: Dashboard calculation verification

This dashboard is intended as a vendor agnostic decision support tool that can be deployed alongside existing CBCT‐ and MR guided ART platforms as well as conventional non adaptive workflows. Rather than replacing commercial online adaptation solutions, the dashboard aggregates geometric, dosimetric, and radiobiological metrics across fractions and across systems to provide quantitative, standardized triggers for when adaptation should be considered. In particular, it is designed to support clinics that primarily deliver static plan treatments (with or without offline replanning) by indicating when accumulated deviations suggest that a re plan or transition to an adaptive workflow may be warranted. To evaluate the efficiency of the dashboard, one fraction with four PTVs and nine OARs took approximately 2 min and 28 s per fraction to calculate. This includes all of the patient specific visuals and calculations described in Section 2.6.

#### 4.1.1 Central point verification

Residuals for central point localization were small across all axes, with mean differences on the order of *< *0.05 mm and maximum deviations *< *0.5 mm (Figure 9). These results indicate that the dashboard reproduces MIM's central point localizations with very high fidelity. The small but statistically significant difference observed along the x‐axis (Table 5) corresponded to a negligible effect size (Cohen's *d *= −0.12, Table 6), confirming that while systematic offsets were detectable, they are unlikely to have clinical or workflow implications.

#### 4.1.2 Dice score verification

**Dice score intrafraction verification**: The intrafraction Dice results showed mean residuals near zero for most structures, with slightly larger differences observed for hollow or highly deformable organs (e.g., bladder, urethra, rectum). These structures are more sensitive to inherent factors like contouring uncertainty, deformation, and filling effects, which can amplify small calculation differences between platforms. The effect size was small (*d *= −0.42), suggesting that the observed differences are not practically meaningful.

**Dice score interfraction verification interfraction**: Dice residuals followed a similar pattern, with the urethra again showing the largest mean difference (−0.49). This is consistent with day‐to‐day anatomical variation and segmentation challenges for small‐volume structures. While a statistically significant difference was detected (Table 5), the effect size was small (*d *= −0.43). Taken together, both intra‐ and interfraction comparisons demonstrate that the dashboard provides consistent overlap metrics, with deviations primarily driven by small‐structure sensitivity rather than systematic miscalculation.

#### 4.1.3 Score card verification

Dose‐based score card comparisons showed strong agreement between platforms. The largest observed mean residual, at D99.9%, was 1.31 Gy. To provide clinical context for this difference, the percent difference was computed for D99.9%: dashboard = 51.29 Gy versus MIM = 52.38 Gy, yielding ∆_%_ = 2.08%, which is below the 5% action level recommended by TG 114.^16^

Percent‐volume and absolute‐volume objectives also showed small overall mean differences (*< *1% or *< *1 (cm^3^) on average). The residual distributions suggest that isolated cases contributed to the maximum deviations, whereas mean residuals remained low.

#### 4.1.4 Radiobiology verification

Radiobiological metrics (*P_I_*, *P*_+_, *P_B_*) showed slightly larger variability than geometric or dosimetric endpoints, with mean residuals up to +1.78% and maximum deviations as high as 8.70% (Figure 13). These differences likely reflect the sensitivity of radiobiological models to small shifts in DVH shape—particularly in the high‐dose region—combined with voxelization and penumbra‐edge sampling effects that influence how dose is assigned near steep gradients, especially for small or boundary‐adjacent structures. Residuals were consistent in magnitude and direction across patients, indicating that the dashboard and MIM are applying the same underlying equations; the observed deviations are therefore better interpreted as consequences of inherent model and DVH sensitivity rather than implementation errors. Since radiobiological endpoints are calculated from the integral DVH, even subtle differences in voxel inclusion near the penumbra can lead to amplified deviations in *P_I_*, *P*_+_, and *P_B_*.

As such, the dashboard provides radiobiology metrics that are consistent in trend and direction, even if small absolute deviations may occur in sensitive cases due to inherent DVH and model sensitivity.

#### 4.1.5 Validation statistics

The summary of mean and maximum absolute differences (Tables 3, 4) highlights that while some metrics reached isolated large residuals (e.g., interfraction Dice 0.83, D99.9% 5.35 Gy, *P_I_* 8.69%), the mean differences were consistently small. This pattern suggests that the largest deviations were outliers rather than systematic biases. Statistical testing confirmed that only three metrics (central point x, Dice intra‐, and Dice interfraction) reached significance, and all corresponding effect sizes were negligible or small (Table 6). From a clinical perspective, this supports the dashboard as a robust and reliable tool for ART monitoring, providing metric calculations that are statistically consistent with MIM and operationally equivalent within the limits of clinical decision thresholds.

#### 4.1.6 Future work

Future expansion of the patient pool will enable statistical comparisons across cohorts and refinement of adaptive triggers. By analyzing metrics across all fractions and multiple patients, robust, data‐driven thresholds for dashboard alerts can be developed. These thresholds should incorporate geometric, dosimetric, and radiobiological deviations, and may ultimately be tailored by disease site or treatment regimen.

Several additional directions warrant further investigation:

- **Site‐specific scoring refinements**: Future studies should examine whether different disease sites require distinct metric weightings or scoring thresholds. For example, overlap metrics may be more critical in H&N treatments, whereas motion‐driven metrics may dominate in prostate cases. Such tailoring could improve the clinical relevance of dashboard scoring across diverse treatment sites.
- **Integration into online adaptive workflows**: Embedding the dashboard into real‐time clinical settings (e.g., MR‐Linac or CBCT‐based ART) would evaluate its feasibility as a decision‐support tool during daily treatment.
- **Prospective clinical validation**: Following retrospective verification, prospective studies are needed to measure the impact of dashboard‐informed adaptation on both clinical outcomes and workflow efficiency.

Collectively, these avenues highlight the potential for the dashboard to evolve from a verification platform into a fully integrated decision‐support system for ART.

## 5 CONCLUSION

### 5.1 Conclusion: Dashboard calculation verification

Anatomical variability during radiotherapy challenges the use of static treatment plans, where fixed margins may result in inadequate target coverage or unnecessary normal tissue exposure. ART seeks to address these uncertainties, but its implementation has been constrained by the lack of practical tools for daily monitoring and decision support.

In this work, we developed and verified a web‐based dashboard for daily dose tracking and visualization, which incorporates geometric, volumetric, dosimetric, and radiobiological metrics. Validation against MIM Maestro demonstrated that mean differences across all metrics were consistently small, with the largest residuals attributable to isolated outliers rather than systematic biases. Statistical testing confirmed that only three metrics (central point x, Dice intrafraction, and Dice interfraction) reached significance, and in all cases the effect sizes were negligible or small. Dose‐volume endpoints, including D99.9%, remained well below the 5% action level recommended by TG‐114,^16^ supporting their clinical interchangeability. Radiobiological metrics showed somewhat larger variability, reflecting inherent sensitivity to DVH sampling in regions of steep dose gradient (penumbra) and voxel inclusion near structure boundaries, but trends were consistent across patients and between platforms.

This verification showed that the dashboard consistently reproduces reference calculations across geometric, volumetric, dosimetric, and radiobiological endpoints. The framework unifies real‐time dose visualization with biologically informed indices, enabling reliable assessment of daily treatment delivery. These findings support the dashboard as a trustworthy research platform and a foundation for future extensions toward ART decision‐making.

## AUTHOR CONTRIBUTIONS

All authors contributed to the conception and design of the study. Chloe DiTusa led the data analysis, dashboard development, and manuscript drafting. All authors reviewed, revised, and approved the final manuscript.

## CONFLICT OF INTEREST STATEMENT

The authors declare no conflicts of interest.

## Supporting information



Supporting information

Supporting information
