# Supplementary material for: Developing a web‐based dashboard for adaptive radiotherapy workflows
Source: J Appl Clin Med Phys. 2026 Apr 6;27(4):e70546. doi: 10.1002/acm2.70546 (PMC13053168; doi:10.1002/acm2.70546)
Supplement: Supplementary file 1 — Supporting information [file ACM2-27-e70546-s002.pdf]

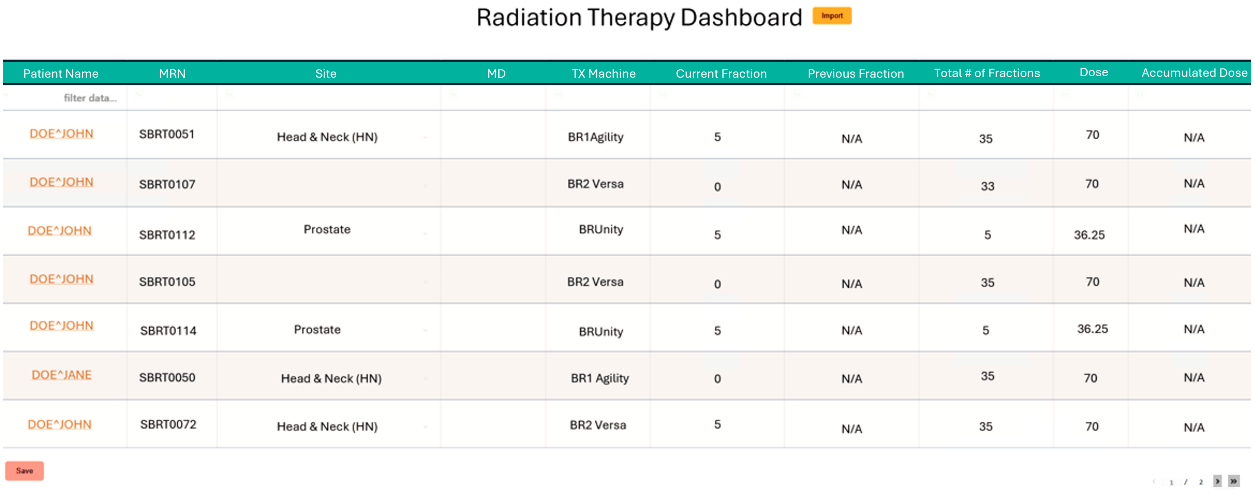

Patient Name	MRN	Site	MD	TX Machine	Current Fraction	Previous Fraction	Total # of Fractions	Dose	Accumulated Dose
filter data...									
DOE^JOHN	SBRT0051	Head & Neck (HN)		BR1Agility	5	N/A	35	70	N/A
DOE^JOHN	SBRT0107			BR2 Versa	0	N/A	33	70	N/A
DOE^JOHN	SBRT0112	Prostate		BRUnity	5	N/A	5	36.25	N/A
DOE^JOHN	SBRT0105			BR2 Versa	0	N/A	35	70	N/A
DOE^JOHN	SBRT0114	Prostate		BRUnity	5	N/A	5	36.25	N/A
DOE^JANE	SBRT0050	Head & Neck (HN)		BR1 Agility	0	N/A	35	70	N/A
DOE^JOHN	SBRT0072	Head & Neck (HN)		BR2 Versa	5	N/A	35	70	N/A

Figure S-1. Dashboard Patient Table summarizing MRN, disease site, treating physician, total prescribed fractions, current fraction, prescription dose, and cumulative score.
