# Supplementary material for: Developing a web‐based dashboard for adaptive radiotherapy workflows
Source: J Appl Clin Med Phys. 2026 Apr 6;27(4):e70546. doi: 10.1002/acm2.70546 (PMC13053168; doi:10.1002/acm2.70546)
Supplement: Supplementary file 2 — Supporting information [file ACM2-27-e70546-s001.pdf]

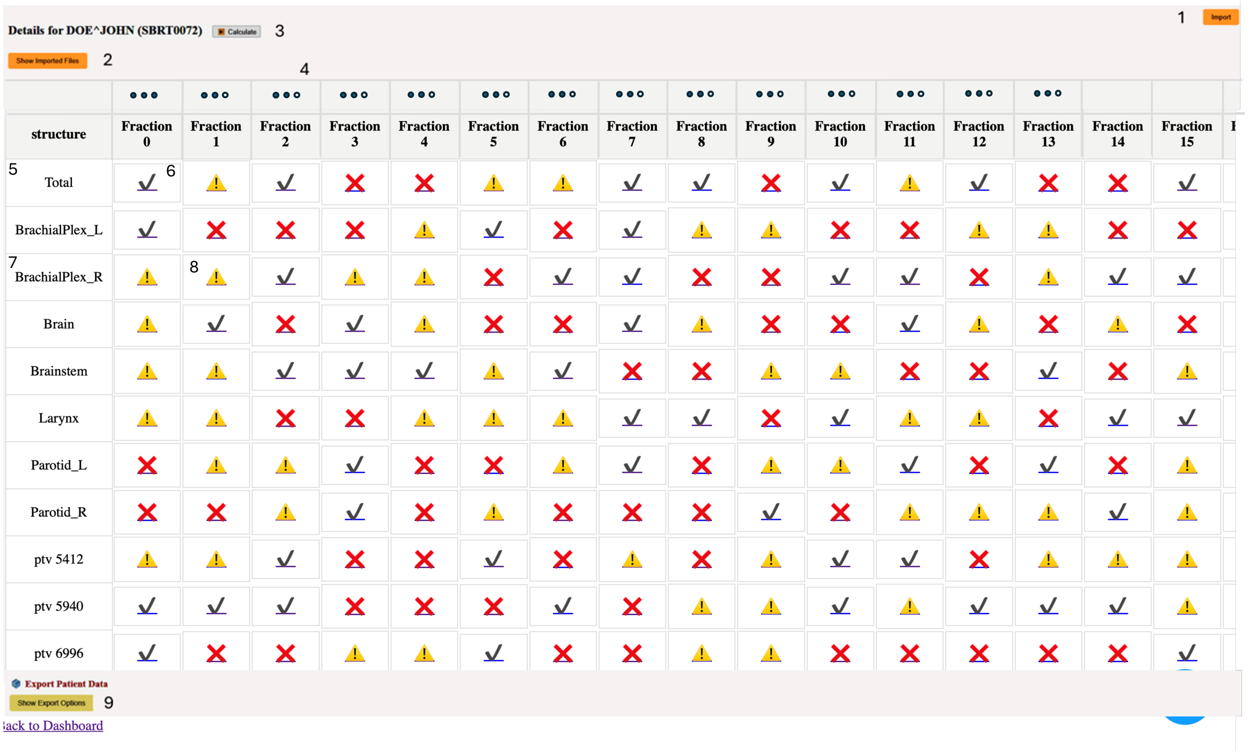

Figure S-2. Fraction-specific scores for an individual patient. The numbered labels (1–9) correspond to the key Dashboard functions described in the text: (1) Import, (2) Show Imported Files, (3) Calculate, (4) file indicators — a compact three-dot indicator above each fraction summarizes the presence of Dose, Structure Set, and Plan files (in that order), (5) Total row, (6) Total status, (7) Structure rows, (8) Structure status indicators, and (9) Export Patient Data.
